# Accumulation of low density lipoprotein associated cholesterol in calcifying vesicle fractions correlates with intimal thickening in thoracic aortas of juvenile rabbits fed a supplemental cholesterol diet

**DOI:** 10.1186/1476-511X-5-25

**Published:** 2006-10-16

**Authors:** Howard HT Hsu, Nathan C Culley

**Affiliations:** 1 Department of Pathology and Laboratory Medicine, Cardiovascular Research Division, University of Kansas Medical Center, Kansas City, KS, 66160, USA; 2 Laboratory Animal Resources, University of Kansas Medical Center, Kansas City, KS, 66160, USA

## Abstract

**Background:**

It has been shown that calcifying vesicles play an important role in aortic calcification and that cholesterol content in the isolated vesicle fraction is increased when rabbits are fed supplemental cholesterol diets. Whether lipoprotein-associated cholesterols and other lipids are also increased in the vesicle fraction and whether the increase correlates with atherosclerosis remain unknown.

**Results:**

Fourteen juvenile male rabbits fed an atherogenic diet containing 0.5% cholesterol and 2% peanut oil for 3 months developed varying degrees of hypercholesterolemia and intimal thickening in the ascending thoracic aorta. The correlation between these two parameters was insignificant, and likely attributable to the use of small numbers of rabbits in this study. Despite this lack of correlation, we demonstrate that the accumulation of cholesterol in calcifying vesicle fractions obtained from the collagenase-digested aorta fragments correlates well with intimal thickening (*r*^2 ^= 0.98, *p *< 0.0001). To a smaller degree, the correlation was also significant between intimal thickening and the cholesterol accumulation in the microsomal and post-vesicle fractions. The cholesterol supplemental diet increased the low density lipoprotein-cholesterol (LDL-C) content in calcifying vesicle fractions by 3-fold but did not affect the triglyceride content. High density lipoprotein-cholesterol (HDL-C) and very low-density lipoprotein cholesterol (VLD-C) were absent in calcifying vesicle fractions.

**Conclusion:**

When limited numbers of rabbits are used, LDL-C accumulation in calcifying vesicle fractions is a better biomarker for atherosclerosis than LDL-C levels in the serum. The close association of LDL-C with calcifying vesicles may play an important role in atherosclerosis and calcification.

## Background

It has been well established from epidemiological studies using a large number of subjects that low-density lipoprotein associated cholesterol (LDL-C) levels in serum correlates with atherosclerosis [1]. However, it is also well known that individual variations exist in response to hypercholesterolemia in animals or humans [2-4]. Some individuals with high serum cholesterol and LDL-C do not suffer atherosclerosis and vice versa is true. This discrepancy leads to findings of other complementary predictors for atherosclerosis and heart failures including C-reactive protein (CRP) [5], troponin (6), and B-type natriuretic peptide [7]. Our previous studies using rabbits as a model revealed that the use of siblings minimized the variations in the correlation between hypercholesterolemia and atherosclerosis when small numbers of rabbits were used [8]. This observation is consistent with the study of human subjects which compared parental and sibling history for the degree of association of hypercholesterolemia with atherosclerosis [9]. Thus, either siblings or a more reliable biomarker for the assessment of atherosclerosis is needed to study the correlation between hypercholesterolemia and atherosclerosis when a limited number of animals are used.

Ultrastructural and *in vitro *mineralization studies have implicated calcifying vesicles with an average size of 200 nm in atherosclerotic calcification [10-18]. We previously demonstrated that a much greater quantity of calcifying vesicles can be isolated from atherosclerotic aortas than those from normal controls. Further, the cholesterol content of calcifying vesicles was substantially higher than normal control vesicles [16]. Although the role of this increase is unclear, the association of cholesterol with calcifying vesicles may play a part in aortic calcification since we found that the addition of cholesterol to calcifying media at 1 mg/ml stimulated mineral deposition by vesicles isolated from normal aortas [16]. In contrast, vesicles from atherosclerotic aortas were not activated by cholesterol unless most of cholesterol molecules in the vesicle fractions were removed by a relatively low centrifugal force. Infrared analyses revealed the removal of cholesterol from the vesicle fraction, thereby sensitizing the vesicles to cholesterol stimulation. These data further suggest that vesicles and the associated cholesterol could play an important role in aortic calcification. The increase in both calcium (Ca) depositing activity and cholesterol content in the vesicle fraction as a result of cholesterol supplementation may be indicative of severe atherosclerosis. In this study we determined whether or not various cholesterol-associated lipoproteins are also present in the calcifying vesicle fraction and whether their accumulations in the fraction correlate with the degrees of intimal thickening, as opposed to the serum lipid levels in hypercholesterolemic rabbits fed the cholesterol supplemental diet.

## Results and discussion

### Variations in lesion accumulation and serum lipids levels in rabbits fed a cholesterol supplemental diet

**Figure 1:**
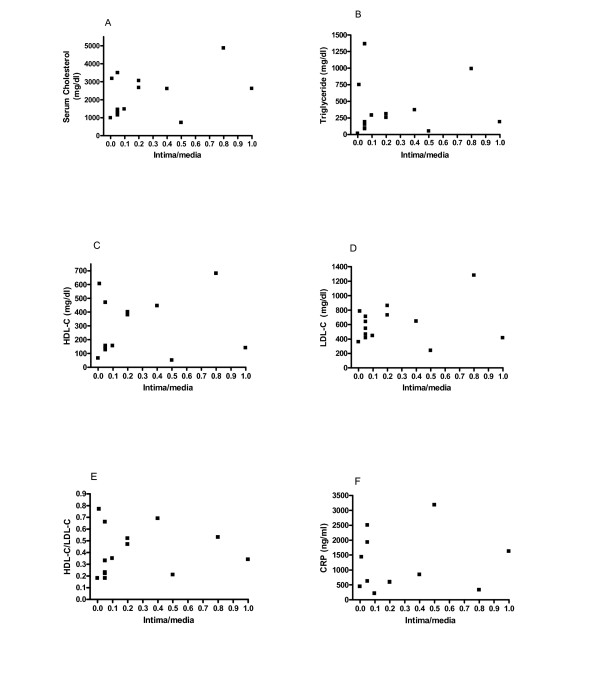
Lack of correlation between lesion development and hyperlipidemia/CRP is due to the use of a limited number of rabbits. Statistical analysis was used to determine the degree of correlation between lesion distributions and serum lipid/CRP levels. A) Total cholesterol, correlation coefficient: *r*^2 ^= 0.25 (*p *= 0.39); B) Triglyceride, *r*^2 ^= 0.001 (*p *= 0.90); C) HDL-C, *r*^2 ^< 0.001 (*p *= 0.92); D) LDL-C, *r*^2 ^= 0.003 (*p *= 0.85); E) the ratio of HDL/LDL, *r*^2 ^< 0.001 (*p *= 0.96); F) CRP, *r*^2 ^= 0.05 (*p *= 0.50). A Prism statistic program (GraphPad Software Inc.) was used for the correlation analysis. Sera were diluted to working ranges of standard curve for cholesterol determination. Fourteen rabbits were fed the cholesterol supplemental diet while 9 rabbits were fed chow diets.

Due to a large variation in the response of human and animals to hypercholesterolemia in the development of atheromatous lesions, it is mandatory to use a large number of human subjects for any epidemiological study. This makes animal study a difficult project because of the prohibitory expenses and time consuming in the use of a large number of rodents such as rabbits with stringent controls of age, gender, and siblings. We first determined whether individual variations in the serum lipid risk factors and biomarkers for atherosclerosis were present in 14 rabbits. In consistence with the established literature [2-4], Table 1 shows large fluctuations in the accumulation of lesions and the blood levels of various risk factors including total cholesterol, HDL-C, LDL-C, triglyceride, and C-reactive protein (CRP) in response to dietary cholesterol supplementation. All nine control rabbits fed a chow diet did not develop lesions and maintained very low levels of blood cholesterol. To determine whether the degree of lesion formation was correlated with various lipid risk factors and biomarkers despite the large fluctuations, we conducted correlation analysis. As shown in Fig. 1, there was a lack of correlation between lesion accumulation and various serum risk factors and biomarkers including total cholesterol, HDL-C, LDL-C, triglyceride, and CRP in response to dietary cholesterol supplementation. These data suggest the large fluctuations are likely attributable to insufficient numbers of rabbits in the study. Our recent report showed that these individual variations can be minimized through the use of siblings [8]. Thus, further identification of a better biomarker or activity, or the use of siblings would be needed to study the progression of lesions in a small sample population setting.

**Table 1:** Varying degrees of intimal thickening, serum lipids, and CRP in rabbits fed cholesterol supplement for 3 months.

Measurements	Chow fed rabbits		Cholesterol diet rabbits		
	M ± SD (n)	Ranges	M ± SD (n)	Ranges	p values

Intima/media	0 (9)	0	0.31 ± 0.37 (14)	0–1.0	<0.0001
Cholesterol (mg/dl)	18 ± 5 (9)	11–25	2200 ± 1196 (14)	725–4865	<0.0001
Triglyceride (mg/dl)	79 ± 46 (9)	13–148	365 ± 396 (14)	15–1365	0.045
HDL (mg/dl)	<5 (9)	<5	282 ± 209 (14)	50–680	<0.0001
LDL (mg/dl)	<10 (9)	<10	610 ± 263 (14)	240–1280	<0.0001
CRP (ng/ml)	230 ± 192 (6)	98–606	1193 ± 948 (12)	212–3184	0.027

The data are means ± S.D. (numbers of rabbits). The *p *values show the statistical significance of differences of each parameter between cholesterol-fed and control rabbits.

### Correlation of cholesterol accumulation in calcifying vesicle fractions with atherosclerosis

We previously reported that both calcifying vesicles [13,14] and co-precipitated cholesterol were increased in rabbit aortas by dietary cholesterol intervention [16]. In this study, we sought to determine whether other lipids were also present in calcifying vesicle fractions and whether the lipid content in vesicle fractions was also affected by the dietary cholesterol treatment.

**Figure 2:**
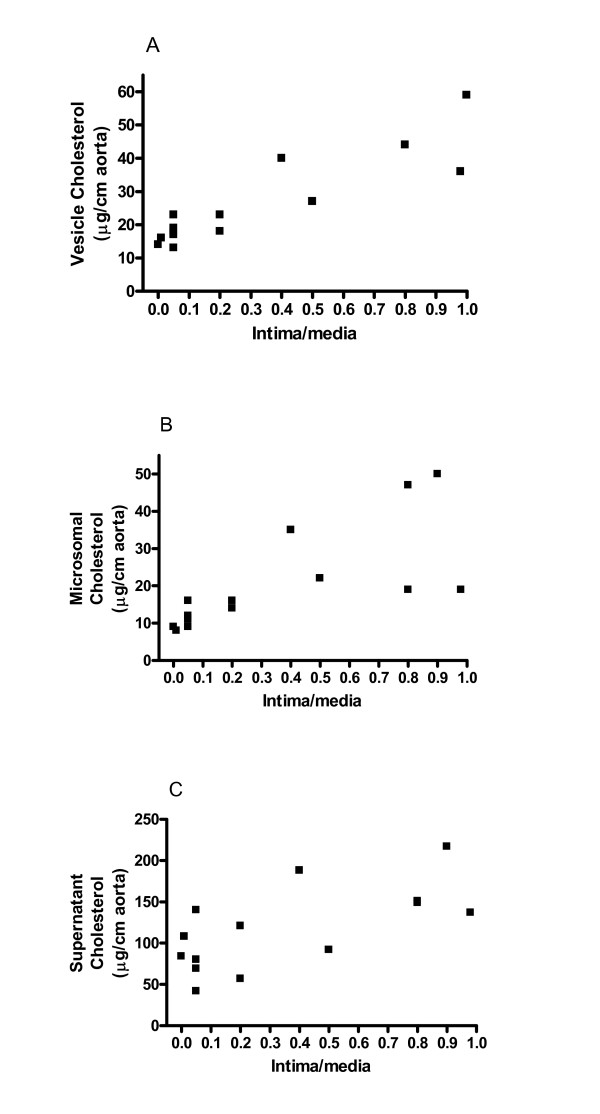
The cholesterol content in various subcellular fractions correlates well with intimal thickening despite the use of limited numbers of rabbits. The cholesterol content in each fraction was run manually using a commercial kit (Thermo DMA Inc.). (A) The cholesterol content in calcifying vesicle fractions; *r*^2 ^= 0.98, *p *< 0.0001. (B) The cholesterol content in microsomal fractions; *r*^2 ^= 0.52, *p *= 0.0036. (C) The cholesterol content in supernatant fractions, *r*^2 ^= 0.45, *p *= 0.009. The content of cholesterol in each fraction was expressed as μg cholesterol/cm of aorta. Fourteen rabbits were fed the cholesterol supplemental diet while 9 rabbits were fed chow diets.

To determine whether cholesterol accumulation in subcellular fractions may play a role in atherogenesis, we studied the correlation between the levels of cholesterol in various subcellular fractions and the degree of lesion formation. As shown in Fig. 2, the cholesterol content of all subcellular fractions was correlated with the degree of atherosclerosis in rabbits fed cholesterol supplements for 3 months. The correlation coefficients for calcifying vesicles, microsomal-mitochondrial, and supernatant fractions were 0.98 (*p *< 0.0001), 0.52 (*p *= 0.0036), and 0.45 (*p *= 0.009), respectively. Comparison of these correlation coefficients and the *p *values among various subcellular fractions revealed a better correlation between cholesterol levels in calcifying vesicle fractions and lesion accumulation than the correlation in other subcellular fractions.

**Figure 3:**
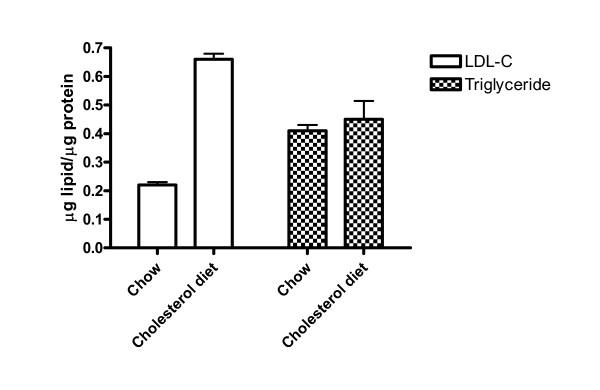
Effects of dietary cholesterol supplementation on the accumulation of LDL-C and triglyceride in calcifying vesicle fractions. The total cholesterol, LDL-C, HDL-C, VLDL-C, and triglyceride in calcifying vesicle fractions were assayed using an automation system in a clinical laboratory. The numbers of chow- and cholesterol-fed rabbits for these experiments were 3 and 4, respectively.

**Figure 4:**
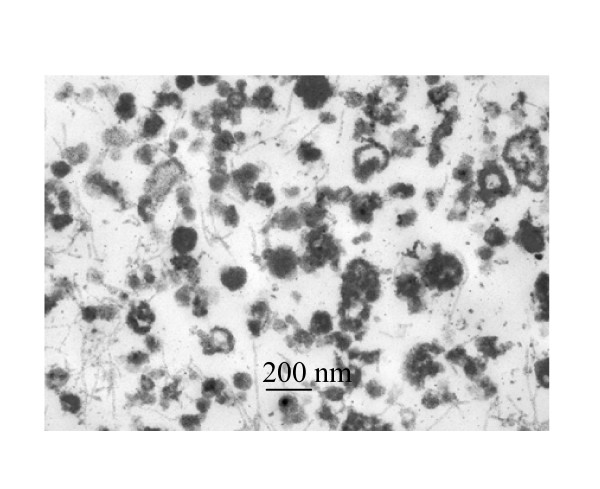
Effect of cholesterol extraction of calcifying vesicles by ethanol on ultrastructural appearance of vesicles. The transmission electron microscopic image of ethanol treated calcifying vesicles was obtained from a cholesterol-fed rabbit. To reduce the number of cholesterol micelles, calcifying vesicle fractions were centrifuged at 250,000 × g for 30 min. To solubilize cholesterol, the resulting pellet was suspended in an aliquot of water and then mixed with alcohol to a final concentration of 60%. The mixture was centrifuged at 10,000 rpm for 15 min in a microfuge and the resulting pellet was processed for electron microscopy. The ultrastructural images did not differ from those obtained from untreated vesicles. The alcohol treatment which presumably solubilized free cholesterol did not alter the LDL-C content in vesicle fraction.

Fig. 3 shows that the vesicle fractions from atherosclerotic aortas contained 0.66 and 0.45 μg LDL-C and triglyceride per μg protein, respectively. All cholesterol molecules in vesicle fractions were associated with LDL since there was no difference in the levels of total cholesterol and LDL-C in the fraction. Within the assay limit, neither HDL-C nor VLDL-C was detected in calcifying vesicle fractions from rabbits fed with or without the supplemental cholesterol diet. The content of LDL-C but not triglyceride in vesicle fractions was significantly increased 3-fold after 3 months of dietary cholesterol intervention. A prolonged overnight incubation of vesicles at 37°C and followed by 60% alcohol extraction of free cholesterol neither altered the appearance of vesicles (Fig. 4) nor the weight ratios of cholesterol to vesicle proteins, suggesting cholesterol was closely associated with vesicles. To determine whether the blood LDL-C could enter aortas as unbound molecules or could co-precipitate with calcifying vesicles, we subjected the blood from atherosclerotic rabbits to the identical centrifugal force for vesicle isolation. As a result, the centrifugation at 250,000 × g for 30 min did not precipitate LDL-C from the blood, suggesting that LDL-C after its entry into aortas was either closely associated with vesicles or co-precipitated with other subcellular organelles.

Altogether, the entry of cholesterol into cells as LDL-C in association with various subcellular fractions, especially, calcifying vesicles, correlates well with the extent of intimal thickening resulting from the cholesterol supplemental diet. In spite of this close correlation, the pathological relevance of the association of LDL with calcifying vesicles and the causal and effect of the two manifestations remain to be established.

## Conclusion

The content of LDL-C in rabbit calcifying vesicles markedly correlates with intimal thickening induced by supplemental cholesterol diet. The association of LDL-C with vesicles may play a role in atherogenesis and aortic calcification.

## Experimental procedures

### Rabbits

Fourteen 4-month-old juvenile New Zealand white male rabbits were fed a chow diet with 0.5% cholesterol and 2% peanut oil for 3 months. Nine control rabbits were fed regular chow diets. Animal care was provided by a staff of veterinarians and technical personnel. The program was monitored in stringency by the Institutional Animal Care and Use Committee at University of Kansas Medical Center.

### Serum lipid analysis

Since serum samples were routinely used for rabbit lipid research, blood samples were collected and allowed to coagulate at room temperature without the addition of heparin or EDTA, which otherwise interferes with calcium determinations in other studies. The blood samples were centrifuged at 1800 rpm for 10 min and the sera were collected for the analyses of lipids and C-reactive protein (CRP). The lipid profile was assayed using a Beckman Synchron automation system in a clinical laboratory. The LDL-C content was directly measured using a standard clinical procedure without the application of the Friedewald equation [19]. The direct measurement procedure which does not require fasting has been included in the National Cholesterol Education Program Working Group on Lipoprotein Measurements [20]. Except for LDL-C, the cholesterol content in subcellular fractions was measured using a commercial kit (Thermo DMA Inc.) CRP was estimated from a commercial kit specific for rabbit serum (Alpha Diagnostic Int.). The hypercholesterolemic sera were diluted to the working ranges of the instruments for each lipid parameter.

### Estimation of lesion accumulation in rabbit thoracic aortas

The lesion accumulation was semi-quantified using a procedure as described previously [16]. Lesions developed fully in the proximal arch of thoracic aortas after 3–4 months of cholesterol feeding and progressed toward the distal section [15,21]. At this stage, about 50% of the middle sections (2.5 cm from the arch joint) were covered with thickened intima. Thus, the middle section was selected as an index of atherosclerosis to allow optimal statistical correlation analysis. To semi-quantify the lesions, the cross sections were stained with H&E, photographed and the images were magnified for photo prints. The weight ratios of the magnified photo images of the thickened intima to the media from the prints were calculated for lesion quantification.

### Isolation of cholesterol-containing subcellular fractions from aortas

Various subcellular fractions of ascending thoracic aortas were isolated according the procedure of Hsu et al. [16]. About 3-inch segments of aortas were dissected and the attached adipose tissues were removed. The segments were immediately submerged in cold phosphate-buffered saline (PBS), minced into fine pieces, washed once with 10 ml PBS by re-suspension and centrifugation. The washed fragments were digested for 3 h at 37°C in a collagenase solution (15 ml/g of tissue) containing 0.1 % of crude collagenase (Boehringer Mannheim, Type B), 0.25 M sucrose, 0.12 M NaCl, 0.01 M KCl, 100 U/ml of penicillin, 1 mg/ml of streptomycin, and 0.02 M TES buffer (N-Tris [hydroxmethyl]-methyl-2-amino-ethanesulfonic acid), pH 7.45. The digests were centrifuged at 800 × g to precipitate cells and undigested debris. The supernatants were then centrifuged at 30,000 × g for 10 min to precipitate mitochondria and microsomes. The supernatants were centrifuged at 250,000 × g for 30 min to precipitate calcifying vesicles. The resulting supernatants were saved and designated as soluble fractions. Each fraction except supernatants was washed twice by mixing the pellets with a buffer containing 0.25 M sucrose and 10 mM Tris-buffered saline, pH 7.6 (STBS) and then followed by centrifugation at the corresponding speeds for each fraction. The resulting pellets were re-suspended in a volume of STBS in proportion to the weight of aortas. The relative purity of each fraction was assayed for enzymatic markers such as succinate dehydrogenase for mitochondria, NADPH (reduced nicotine amide adenine dinucleotide phosphate)-cytochrome C reductase for microsomes, and NTP (nucleoside triphosphate) was run manually using a commercial kit (Thermo DMA Inc.) pyrophosphohydrolase and ATPase for calcifying vesicles according to a previous procedure [13]. The resultant suspensions yielded a protein concentration of about 0.5–1 mg/ml.

## Competing interests

The author(s) declare that they have no competing interests.

## Authors' contributions

Howard H.T. Hsu, Ph.D. was responsible for the experimental designs, data interpretations, and execution of experiments. Nathan C. Culley, D.V. M. played a major role in icterus and atherosclerosis determinations, welfare and sacrifice of the rabbits, and collections of serum and aortas.
